# Analysis of Interleukin-1 Signaling Alterations of Colon Adenocarcinoma Identified Implications for Immunotherapy

**DOI:** 10.3389/fimmu.2021.665002

**Published:** 2021-07-23

**Authors:** Xiaogang Zhou, Yu Liu, Jing Xiang, Yuntao Wang, Qiqian Wang, Jianling Xia, Yunfei Chen, Yifeng Bai

**Affiliations:** ^1^ Department of Gastrointestinal Surgery, Sichuan Provincial People’s Hospital, University of Electronic Science and Technology of China, Chengdu, China; ^2^ Department of Oncology, Sichuan Provincial People’s Hospital, University of Electronic Science and Technology of China, Chengdu, China; ^3^ Department of Outpatient, Sichuan Provincial People’s Hospital, University of Electronic Science and Technology of China, Chengdu, China; ^4^ Department of Oncology, The Second Clinical Medical College, The Fifth People’s Hospital affiliated to Chengdu University of Traditional Chinese Medicine, Chengdu, China; ^5^ The Third Department of Hepatobiliary Surgery and Organ Transplant Center, Sichuan Provincial People’s Hospital, University of Electronic Science and Technology of China, Chengdu, China

**Keywords:** immune checkpoint inhibitors, colon adenocarcinoma, tumor microenvironment, IL-1, prognosis

## Abstract

Immune checkpoint inhibitors (ICIs) have made breakthrough progress in the treatment of various malignant tumors. However, only some patients receiving ICIs obtain long-lasting clinical effects, and some patients still do not achieve remission. Improving the treatment benefits of this part of the population has become a concern of clinicians. IL-1 signaling plays an important role in the tumor microenvironment (TME). However, the relationship between the IL-1 signaling mutation status and the prognosis of colon adenocarcinoma (COAD) patients receiving ICIs has not been reported. We downloaded the data of a COAD cohort receiving ICIs, including prognostic data and mutation data. Additionally, we downloaded the data of a COAD cohort from The Cancer Genome Atlas (TCGA) database, including clinical data, expression data and mutation data. Gene set enrichment analysis (GSEA) was used to assess differences in the activity of some key physiological pathways between the IL-1 signaling mutated-type (IL-1-MT) and IL-1 signaling wild-type (IL-1-WT) groups. The CIBERSORT algorithm was used to evaluate the contents of immune cells in the TME of COAD patients. The multivariate Cox regression model results suggested that IL-1-MT can be used as an independent predictor of a better prognosis in COAD patients receiving ICIs (P = 0.03, HR = 0.269, 95% CI: 0.082-0.883). Additionally, IL-1-MT COAD patients had significantly longer overall survival (OS) (log-rank P = 0.015). CIBERSORT analysis showed that the IL-1-MT group had high infiltration levels of activated dendritic cells (DCs), M1 macrophages, neutrophils, activated natural killer (NK) cells, activated CD4+ memory T cells and CD8+ T cells. Similarly, the IL-1-MT group had significantly upregulated immunogenicity, including in terms of the tumor mutation burden (TMB), neoantigen load (NAL) and number of mutations in DNA damage repair (DDR) signaling. GSEA showed that the IL-1-MT group was highly enriched in the immune response and proinflammatory mediators. Additionally, the expression levels of immune-related genes, immune checkpoint molecules and immune-related signatures were significantly higher in the IL-1-MT group than in the IL-1-WT group. IL-1-MT may be an independent predictor of a good prognosis in COAD patients receiving ICIs, with significantly longer OS in IL-1-MT COAD patients. Additionally, IL-1-MT was associated with significantly increased immunogenicity, activated immune cell and inflammatory mediator levels and immune response-related scores.

## Introduction

Immune checkpoint inhibitors (ICIs) have made breakthrough progress in the treatment of various malignant tumors (1–3). Recent studies have shown that colorectal cancer (CRC) patients who benefit from ICIs are mainly those with high mutation burden and mismatch repair deficiency (dMMR), and this population accounts for only approximately 5% of metastatic CRC (4). Therefore, only some patients receiving ICIs obtain long-lasting clinical effects (5, 6), and some patients still do not achieve remission. Improving the treatment benefits of this part of the population has become a concern of clinicians.

Various potential biomarkers have been found in colon adenocarcinoma (COAD) patients receiving immunotherapy, such as microsatellite instability (MSI), programmed cell death-ligand 1 (PD-L1) expression, tumor mutation burden (TMB) and BRAF and KRAS gene mutation status (6). However, the effects of the above biomarkers are still limited. For example, the dMMR/microsatellite instability high (MSI-H) COAD population is considered to derive the most benefits from ICI treatment, but the effective rate is only 30%-40% (7), and this subset only accounts for a small part of the COAD population. The heterogeneity of PD-L1 expression in time and space is related to differences in detection methods. Additionally, some patients with a low TMB can also respond to immunotherapy, and patients with a high TMB may not show good immunotherapy efficacy (8). Therefore, finding new markers to predict the efficacy of ICIs in COAD patients has become an important challenge.

There are certain correlations and influences between specific mutations or pathway mutations and ICI efficacy markers (9, 10). Mutations in DNA repair pathways are associated with better clinical benefits for patients with multiple tumors after receiving immunotherapy (10). Additionally, SERPINB3 or SERPINB4 mutations are associated with good prognosis in melanoma patients treated with cytotoxic T lymphocyte-associated protein-4 (CTLA-4) blockade (11). TET mutations are associated with higher objective response rates (ORRs), favorable clinical benefits and prolonged progression-free survival (PFS) and overall survival (OS) in pan-cancer cohorts treated with ICIs (PD-(L)1 and/or CTLA-4) (12). The role of interleukins in the antitumor immune response has received increasing attention (13, 14). Previous evidence has confirmed that interleukin-1 (IL-1) promotes the expression of cyclooxygenase-2 in COAD and can also increase the level of cyclooxygenase-2 in COAD cells (15, 16). Cyclooxygenase-2 is involved in the occurrence, development, tumor angiogenesis and metastasis of COAD. Moreover, IL-1 can increase the secretion of matrix metalloproteinases and vascular endothelial growth factor (VEGF) and promote the adhesion of endothelial cells, thus promoting the occurrence and progression of COAD (17). It has been reported that IL-1 family proteins, such as IL-1α, IL-1β and IL-18, may play distinct roles in immune responses during infections and inflammatory diseases (18). IL-1R transduces signals through myeloid differentiation factor 88 (MyD88), which triggers a series of events, leading to the expression of inflammatory genes and the recruitment of immune cells (19, 20). Additionally, studies have indicated that the secretion of IL-1 and other cytokines by monocytes, macrophages, cancer cells and fibroblasts contributes to the formation of tumor-related immunosuppression, which may also explain why IL-1 leads to the development of COAD (21).

However, in COAD, the impact of IL-1 pathway mutations on the clinical prognosis of immunotherapy remains unclear. Hence, in this study, we explored the association between the mutated IL-1 signaling status and the prognosis of COAD patients receiving ICIs and sought to illustrate the potential mechanism between the mutated IL-1 signaling status and the prognosis of patients treated with immunotherapy from the perspective of the immune microenvironment.

## Methods

### Clinical Sample and Group Definition

To explore the impact of IL-1 signaling mutated-type (IL-1-MT) on the prognosis of COAD patients treated with immunotherapy, we downloaded the mutation and clinical data of an ICI-treated COAD cohort (22). The immunotherapy regimen for this cohort was PD-(L)1 or combination with CTLA-4 inhibitors. Additionally, we used the “TCGAbiolinks” R package (23) and downloaded the COAD expression data, mutation data and clinical data from TCGA (https://portal.gdc.cancer.gov/). Nonsynonymous mutation data were used to quantify the status of each patient’s mutations in IL-1 signaling. If a patient had no mutations in IL-1 signaling, their status was defined as IL-1 signaling wild-type (IL-1-WT); otherwise, their status was defined as IL-1-MT.

### Data Preprocessing

According to the definition of the TMB in published literature (24), we evaluated the TMB in the TCGA-COAD cohort. Additionally, the neoantigen load (NAL), immune-related genes and immune-related signatures/scores were collected from published studies (25, 26). We downloaded eight gene sets of DNA damage repair (DDR) signaling from the Molecular Signatures Database (MSigDB) and merged them into one DDR gene set (27). Next, we used these nine gene sets to analyze the number of mutations in each patient’s DDR-related pathways. The CIBERSORT algorithm (28) was used to evaluate the proportions of 22 immune cells (https://cibersort.stanford.edu/index.php). Additionally, the ClusterProfiler R package (29) and pathways from the Gene Ontology (GO), Kyoto Encyclopedia of Genes and Genomes (KEGG) and REACTOME databases were used in gene set enrichment analysis (GSEA).

### Statistical Analysis

In the ICI-treated COAD cohort, univariate and multivariate Cox regression models and Kaplan-Meier (KM) curves were used to analyze the influence of the IL-1 pathway mutation status and clinical characteristics on the survival of COAD patients. The Mann-Whitney U test was used to compare differences in continuous variables between the two groups (IL-1-MT and IL-1-WT). Fisher’s exact test was used to compare differences in the categorical variables between the two groups (IL-1-MT and IL-1-WT). Log-rank P was used to reflect significant differences. P < 0.05 was considered significantly different, and all analyses in this study were completed using R software (version 3.6.3).

## Results

### IL-1-MT Can Be Used as an Independent Predictor of Better Prognosis in COAD Patients Receiving ICIs

**Figure 1:**
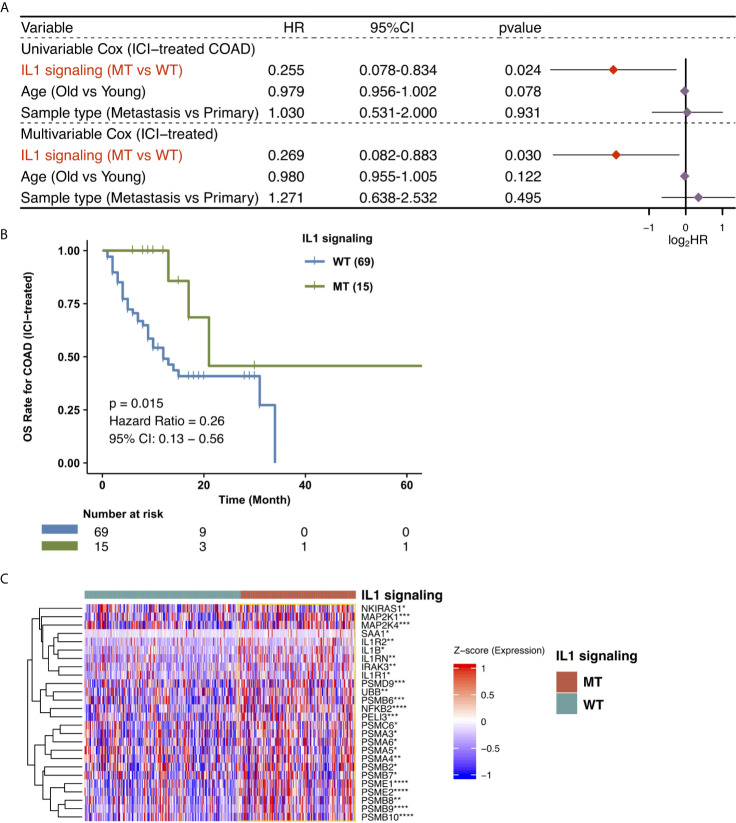
Predictive values of clinical characteristics and the IL-1 signaling mutation status on ICI outcomes. **(A)** Forest plot of the results of the univariate Cox and multivariable Cox regression models. In the univariate Cox regression analysis, the factors with a p value below 0.05 were IL-1 signaling MT. Multivariate Cox regression analysis showed that the IL-1 signaling mutation status was an independent predictor of ICI therapy in COAD patients. The main part of the forest plot presents the risk ratio (HR) and 95% confidence interval (95% CI), where red dots indicate P < 0.05. The HR indicates predictors of favorable (HR < 1) or poor (HR > 1) OS. **(B)** Kaplan-Meier survival curves for the OS of 84 COAD patients in the ICI-treated cohort. We performed KM survival analysis on different subgroups of patients (IL-1 signaling mutation status). **(C)** Heatmap for the expression of markers related to IL1 signaling. (*P < 0.05; **P < 0.01; ***P < 0.001; ****P < 0.0001; Mann-Whitney U test).

To explore the effect of the mutated IL-1 signaling status on the prognosis of COAD patients receiving ICIs, we downloaded the data of a cohort of COAD patients treated with ICIs from the CBioPortal webpage (https://www.cbioportal.org/). The IL-1 signaling gene set from MSigDB was collected, and the number of gene mutations in the IL-1 signaling pathway in each patient was calculated. Then, the univariate Cox regression model was used for subsequent analysis. We found that age and sample type were not significantly associated with the immunotherapy prognosis (P > 0.05), while IL-1-MT COAD patients treated with immunotherapy were associated with better prognosis (P = 0.024, hazard ratio (HR) = 0.255, 95% confidence interval (CI): 0.078-0.834; **Figure 1A**). The multivariate Cox regression model was used to analyze whether IL-1-MT could be used as an independent predictor of the prognosis of COAD patients. The results showed that only IL-1-MT could be used as an independent predictor of good prognosis in COAD patients receiving ICIs (P = 0.03, HR = 0.269, 95% CI: 0.082-0.883; **Figure 1A**). Next, the KM curve also showed that COAD patients who had mutations in IL-1 signaling had significantly prolonged OS compared to COAD patients who did not have mutations in IL-1 signaling (**Figure 1B**, log-rank P = 0.015). Moreover, we compared the differential expression of genes related IL-1 signaling pathway between IL1-MT and IL1-WT groups. We found that IL1-MT COAD patients had significantly higher expression levels of markers related to the IL-1 signaling pathway compared with IL1-WT COAD patients (**Figure 1C**).

### The Relationships Between IL-1-MT and Mutation Characteristics and Clinical Characteristics

**Figure 2:**
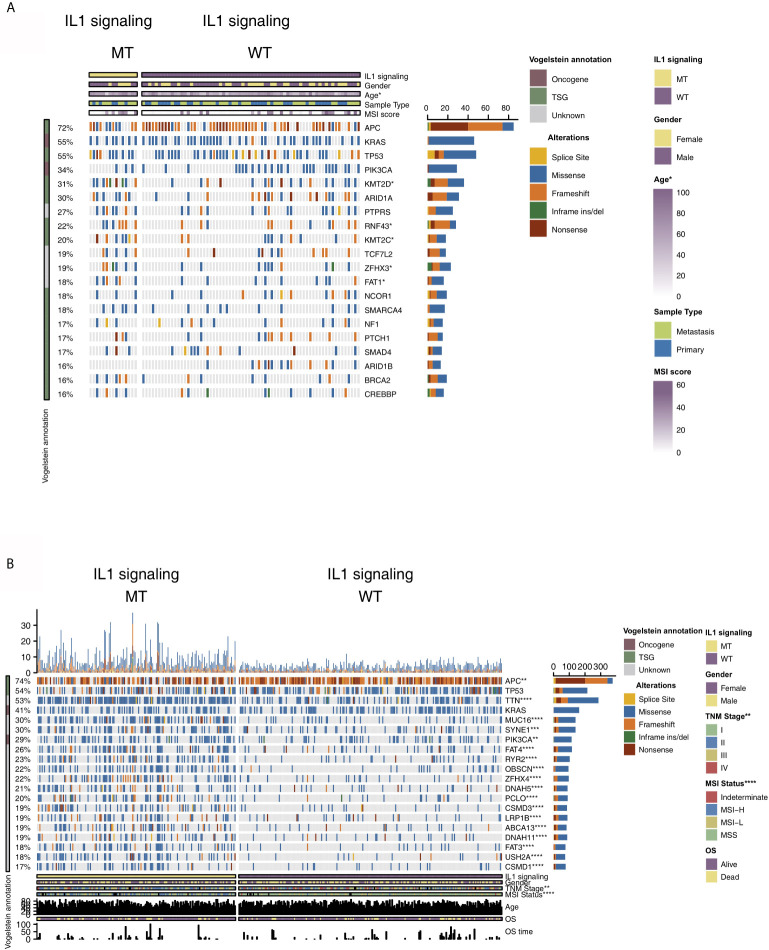
Genomic profiles of COAD patients in the ICI-treated **(A)** and TCGA-COAD **(B)** cohorts. The top 20 genes with the highest mutation frequencies and the corresponding clinical information are shown in the figure. The top five genes with the highest mutation frequencies in the ICI-treated cohort were APC (72%), KRAS (55%), TP53 (55%), PIK3CA (34%) and KMT2D (31%). The top five genes with the highest mutation frequencies in the TCGA-COAD cohort were APC (74%), TP53 (54%), TTN (53%), KRAS (41%) and MUC16 (30%). For the mutation type, yellow indicates splice site mutations, blue indicates missense mutations, orange indicates frameshift mutations, green indicates inframe indel mutations and brown indicates nonsense mutations. The IL-1 signaling mutation status, sex, age, sample type and MSI score are shown as patient annotations (the upper/lower bar plot). The left bar plot marks the mutation rate of each gene. (*P < 0.05, **P < 0.01, ***P < 0.001 and ****P < 0.0001; Fisher’s exact test).

**Figure 3:**
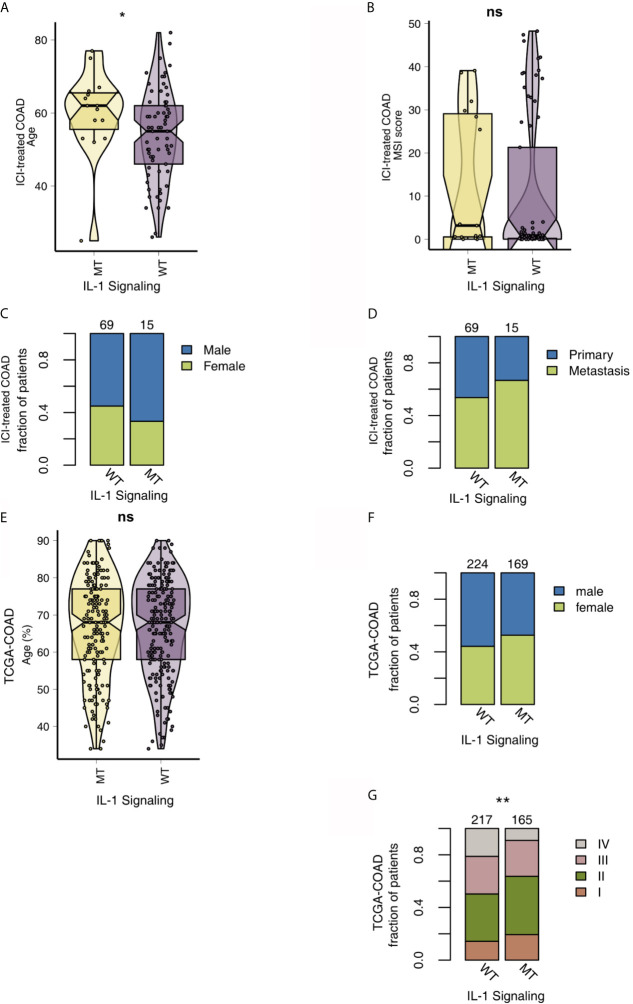
Comparison of clinical characteristics between IL-1-MT and IL-1-WT tumors. **(A)** Comparison of age between IL-1-MT and IL-1-WT tumors in the ICI-treated COAD cohort. **(B)** Comparison of the MSI score between IL-1-MT and IL-1-WT tumors in the ICI-treated COAD cohort. **(C)** Comparison of the sex proportions between IL-1-MT and IL-1-WT tumors in the ICI-treated COAD cohort. **(D)** Comparison of the sample type proportions between IL-1-MT and IL-1-WT tumors in the ICI-treated COAD cohort. **(E)** Comparison of age between IL-1-MT and IL-1-WT tumors in the TCGA-COAD cohort. **(F)** Comparison of the sex proportions between IL-1-MT and IL-1-WT tumors in the TCGA-COAD cohort. **(G)** Correlation analysis of the clinical stage proportions between IL-1-MT and IL-1-WT tumors in the TCGA-COAD cohort. (*P < 0.05; **P < 0.01; ns, not significant).

To analyze the relationship between IL-1-MT and the mutation characteristics of COAD patients, we analyzed nonsynonymous mutations in the ICI-treated COAD cohort and TCGA-COAD cohort. In the ICI-treated COAD cohort (**Figure 2A**), the genes with the top 20 mutation frequencies were APC, KRAS, TP53, PIK3CA, KMT2D, ARID1A, PTPRS, RNF43, KMT2C, TCF7L2, ZFHX3, FAT1, NCOR1, SMARCA4, NF1, PTCH1, SMAD4, ARID1B, BRCA2 and CREBBP. Most of these genes are tumor suppressor genes (TSGs), followed by oncogenes and unknown genes. Except for APC, KMT2D, ARID1A and RNF43, most of the mutation types were nonsense and frameshift mutations, and most of the remaining gene mutation types were missense mutations. Additionally, some genes had significantly increased mutation frequencies in the IL-1-MT group compared with the IL-1-WT group, such as KMT2D (60% *vs.* 25%), RNF43 (47% *vs.* 16%), KMT2D (47% *vs.* 15%), ZFHX3 (40% *vs.* 15%) and FAT1 (40% *vs.* 14%) (**Figure 2A**). In the TCGA-COAD cohort, the genes with the top 20 nonsynonymous somatic mutations were APC, TP53, TTN, KRAS, MUC16, SYNE1, PIK3CA, FAT4, RYR2, OBSCN, ZFHX4, DNAH5, PCLO, CSMD3, LRP1B, ABCA13, DNAH11, FAT3, USH2A and CSMD1. Because the detection method used for the TCGA-COAD cohort was whole-exome sequencing (WES), more somatic mutation data were available for this cohort. We found that the mutation frequencies of the top 20 genes in the IL-1-MT group were significantly higher than those in the IL-1-WT group (all P < 0.05; **Figure 2B**). Among these genes, only KRAS and PIK3CA are oncogenes. The mutation sites of IL-1 family genes that recruit MyD88, IRAK4 and TRAF6 were visualized in **Figure S1**. Next, we compared the differences in clinical characteristics between the IL-1-MT and IL-1-WT groups. In the ICI-treated COAD cohort, the IL-1-MT group contained older patients (**Figure 3A**; P < 0.05). However, in terms of the MSI score, sex ratio and origin of samples, we did not find significant differences between the two groups (**Figures 3B–D**; all P > 0.05). In the TCGA-COAD cohort, there were no significant differences between the IL-1-MT group and the IL-1-WT group in age or sex ratio (**Figures 3E, F**). Additionally, the IL-MT group had a higher proportion of early clinical patients (**Figure 3G**).

### Immune Microenvironment Under Different IL-1 Signaling Mutation Statuses

**Figure 4:**
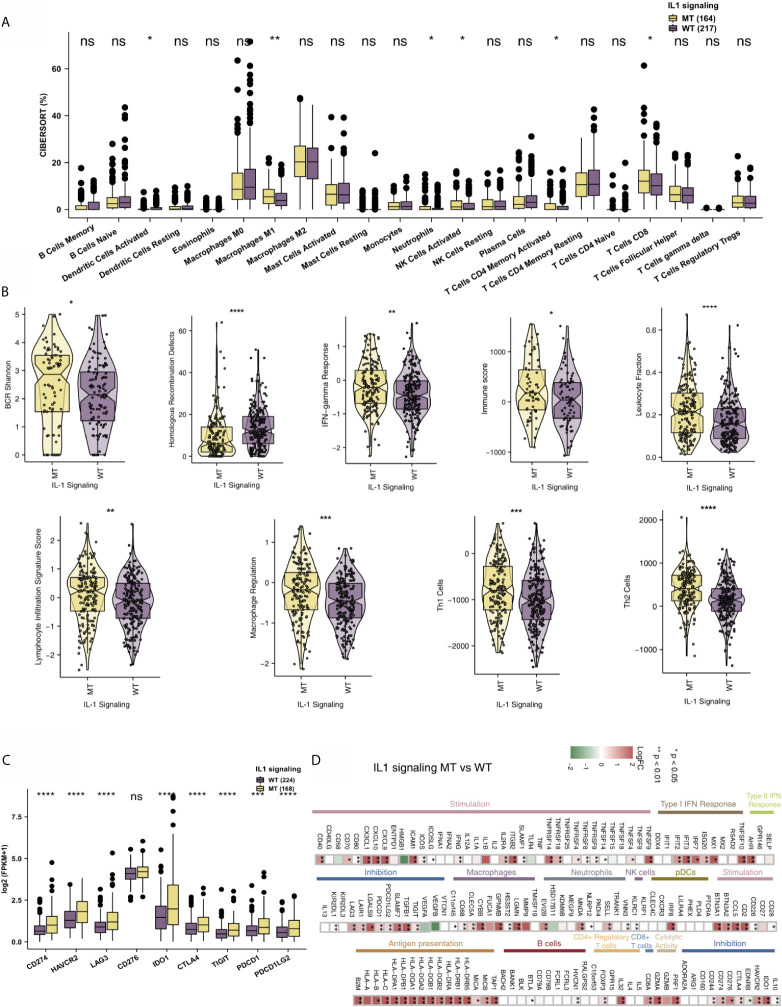
IL-1-MT COAD is associated with an inflammatory TME. **(A)** Comparison of the fractions of 22 immune cells between IL-1-MT and IL-1-WT tumors in the TCGA-COAD cohort. **(B)** Comparison of the immune-related signatures between IL-1-MT and IL-1-WT tumors in the TCGA-COAD cohort. **(C)** Comparison of the expression levels of immune checkpoint molecules between IL-1-MT and IL-1-WT tumors in the TCGA-COAD cohort. **(D)** Heatmap depicting the mean differences in immune-related gene mRNA expression between IL-1-MT and IL-1-WT tumors across different cancer types. The x-axis of the heatmap indicates different cancer types, and the y-axis indicates gene names. Each square represents the fold change or difference of each indicated immune-related gene between IL-1-MT and IL-1-WT tumors in each cancer type. Red indicates upregulation, while blue indicates downregulation (*P < 0.05; **P < 0.01; ***P < 0.001; ****P < 0.0001; ns, not significant; Mann-Whitney U test).

**Figure 5:**
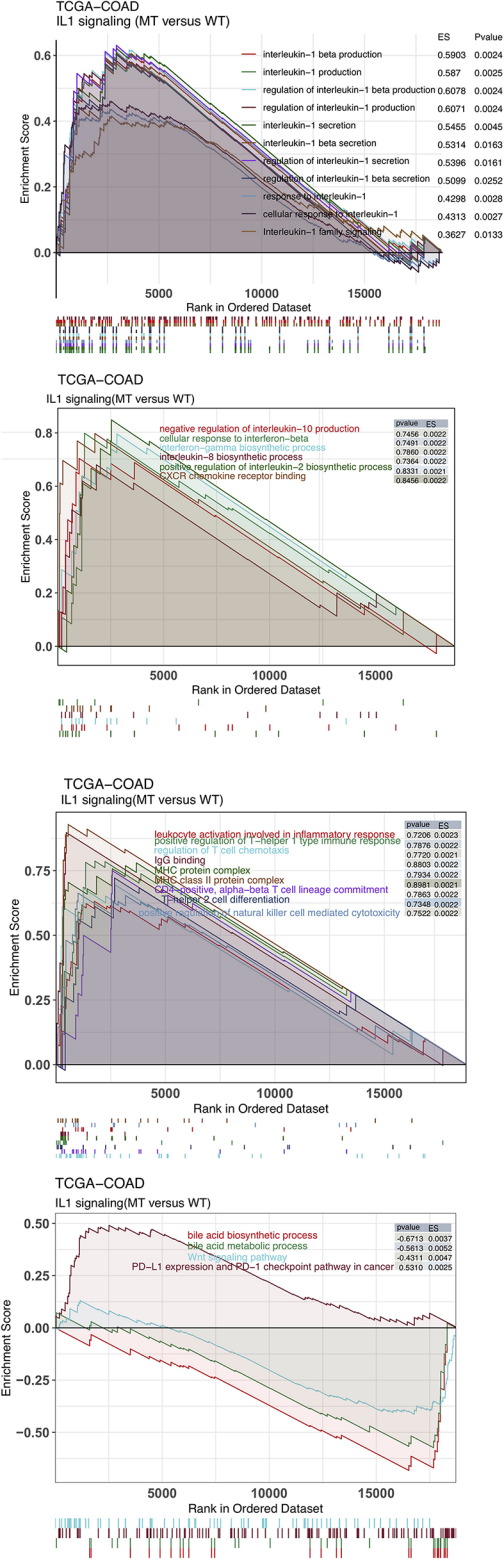
The results of gene set enrichment analysis. The color of the curve corresponds to the font color of the pathway. GSEA of hallmark gene sets downloaded from the Molecular Signatures Database (MSigDB). Each run was performed with 1000 permutations. Enrichment results with significant differences (P < 0.05) between IL-1-MT and IL-1-WT tumors are shown.

The immune microenvironment is one of the key factors that affects whether patients receive ICIs, and it is based on the perspectives of tumor-infiltrating lymphocytes (TILs), immune-related signatures, immune checkpoint molecules and immune-related genes. First, the CIBERSORT algorithm evaluated the proportions of 22 immune cells based on the expression data of COAD patients. **Figure 4A** shows the differences in the ratios of 22 immune cells between the IL-1-WT group and IL-1-MT group. We found that functionally active TILs were significantly enriched in the tumor microenvironment (TME) in patients with IL-1-MT COAD, such as activated dendritic cells (DCs), M1 macrophages, neutrophils, activated natural killer (NK) cells, activated CD4+ memory T cells and CD8+ T cells. In addition to the proportion of immune cell infiltration, immune-related signature analysis showed that IL-1-MT COAD patients had a higher score/activity related to the immune response (**Figures 4B**), such as BCR Shannon, homologous recombination defects, IFN-gamma response, immune score, leukocyte fraction, lymphocyte infiltration signature score, macrophage regulation, Th1 cells and Th2 cells. Similarly, the expression of immune checkpoint molecules, such as CD274 (PD-L1), HAVCR2, LAG3, IDO1, CTLA4, TIGIT, PDCD1 and PDCD1LG2, in the IL-1-MT group was significantly higher than that in the IL-1-WT group (**Figure 4C**). Immune-related genes, such as proinflammatory factors (IFNG, TNFSF10, TNFSF9, TNFSF4, TNFSF14, TNFRSF9, TNFRSF8, TNFRSF4, TNFRSF18 and TNFRSF14), chemokines (CXCL9, CXCL10 and CX3CL1), cytotoxic function markers (PRF1, GZ and CD8A) and antigen processing and presentation markers (TAP1, MICB, MICA, HLA-DRB5, HLA-DRB1, HLA-DRA, HLA-DQB2, HLA-DQB1, HLA-DQA1, HLA-DQA2, HLA-DPB1, HLA- DPA1, HLA-C, HLA-B and B2M), were also enriched in the IL-1-MT group (**Figure 4D**). Additionally, we found that the expression of IL-1 family genes with proinflammatory activity was significantly higher in the IL-1-MT group than the IL-1-WT group (**Figure S2**, **3**). The GSEA results showed the activity of IL1 related pathways significantly higher in the IL1-MT group compared to the IL1-WT group (**Figure 5**). Also, the activities of chemokines and other signaling pathways, such as CXCR chemokine receptor binding, positive regulation of interleukin-2 biosynthetic process, interferon-gamma biosynthetic process, cellular response to interferon-beta, negative regulation of interleukin-10 production and interleukin-8 biosynthetic process, were significantly activated in the IL-1-MT group (**Figure 5**). Additionally, the enrichment scores of some signaling pathways involved in the immune response were significantly higher in the IL-1-MT group than in the IL-1-WT group, such as MHC class II protein complex, IgG binding, positive regulation of T-helper 1 type immune response, CD4-positive, alpha-beta T cell lineage commitment, regulation of T cell chemotaxis, positive regulation of natural killer cell-mediated cytotoxicity, T-helper 2 cell differentiation, leukocyte activation involved in inflammatory response and MHC protein complex. In contrast, some signaling pathway activities related to immune depletion, such as PD-L1 expression and the PD-1 checkpoint pathway in cancer, Wnt signaling pathway, bile acid metabolic process and bile acid biosynthetic process, were significantly activated in the IL-1-WT group (**Figure 5**).

### Differences in Immunogenicity Under Different IL-1 Signaling Mutation Status

**Figure 6:**
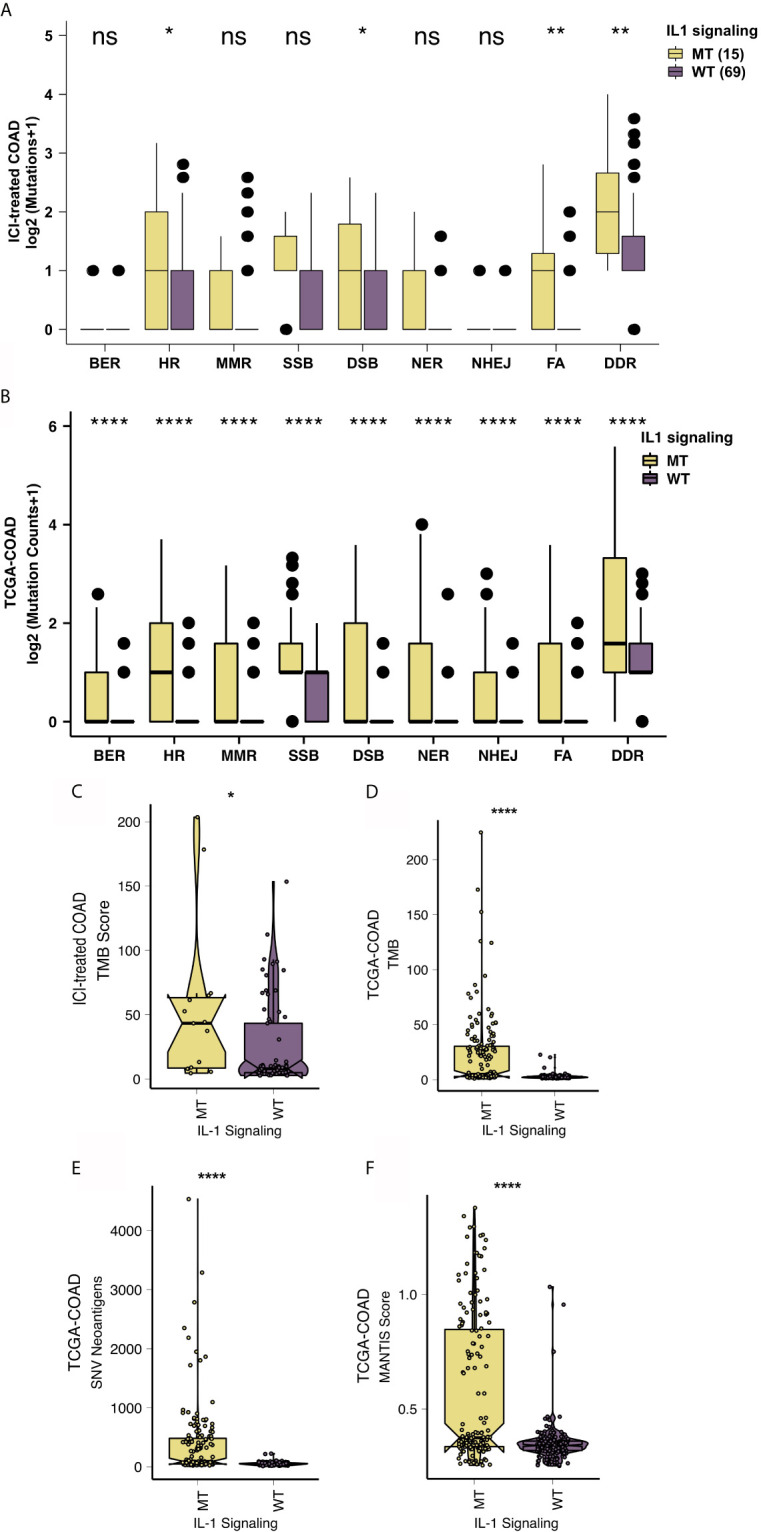
IL-1-MT COAD is associated with enhanced tumor immunogenicity. Comparison of the mutation counts of nine DNA damage-related signaling pathways between IL-1-MT and IL-1-WT tumors in the ICI-treated COAD **(A)** and TCGA-COAD **(B)** cohorts. Comparison of the TMB between IL-1-MT and IL-1-WT tumors in the ICI-treated COAD **(C)** and TCGA-COAD **(D)** cohorts. Comparison of the NAL between IL-1-MT and IL-1-WT tumors in the TCGA-COAD cohort **(E)**. Comparison of the MANTIS score between IL-1-MT and IL-1-WT tumors in the TCGA-COAD cohort **(F)**. (*P < 0.05; **P < 0.01; ****P < 0.0001; ns, not significant; Mann-Whitney U test).

The level of immunogenicity is one of the important factors that affects patients’ acceptance of ICIs. Therefore, we started from the perspectives of the TMB, NAL, the MANTIS score and DDR pathway mutations. The DDR pathway gene set from MSigDB was used to count the DDR pathway mutations of each patient. In the ICI-treated COAD cohort, IL-1-MT COAD patients had a significantly increased number of DDR pathway mutations. Additionally, patients with IL-1-MT COAD had more mutations in the homologous recombination (HR), double-strand break (DSB) and Fanconi anemia (FA) pathways (**Figure 6A**). Similarly, in the TCGA-COAD cohort, IL-1-MT COAD patients had significantly increased gene mutations in all DDR-related pathways (**Figure 6B**; all P < 0.05). Additionally, in both the ICI-treated COAD cohort and TCGA-COAD cohort, the IL-1-MT group had a higher TMB than the IL-1-WT group (**Figures 6C, D**; all P < 0.05). In the TCGA-COAD cohort, the IL-1-MT group had a significantly increased NAL compared with the IL-1-WT group (**Figure 6E**, P < 0.05). The MANTIS score is used as a marker to measure the MSI status. The MSI phenotypes of samples with higher MANTIS scores are closer to MSI-H. **Figure 6F** shows that the MANTIS score in the IL-1-MT group was significantly higher than that in the IL-1-WT group (P < 0.05).

## Discussion

In this study, we used the prognostic and mutation data of COAD patients treated with ICIs and explored the influence of the IL-1 signaling mutation status on the prognosis of patients treated with immunotherapy. The results of univariate and multivariate Cox regression model analyses showed that IL-1-MT can be used as an independent predictor of good prognosis for COAD patients receiving ICIs. Additionally, compared with IL-1-WT COAD patients, IL-1-MT COAD patients had significantly improved OS. Two-way regulation can occur between tumor cells and immune cells in the TME. Tumor cells recruit and regulate the behavior of immune cells by secreting growth factors and cytokines, and the interaction between tumor cells and immune cells can also extend to the body. The balanced state of the cell mobilizes the resources inside and outside the cell, creates a TME suitable for its own growth and affects the response of tumor cells to immunotherapy (6, 30). We found that the TME of IL-1-MT COAD patients has a high infiltration level of activated immune cells. Additionally, the IL-1-MT group had significantly increased expression levels of immune checkpoint molecules, proinflammatory-related genes, antigen presentation-related genes, chemokine-related genes and cytotoxicity-related genes. IL-1-MT COAD patients had enhanced immunogenicity, which mainly manifested as an increased TMB and NAL and an increased number of DDR pathway mutations.

The activated immune cells enriched in the immune microenvironment in the IL-1-MT group may be a potential mechanism for improved prognosis after ICI treatment. Studies have shown that the presence of highly infiltrated TILs in tumors, especially CD4+ T cells and CD8+ T cells, is related to the good clinical prognosis of patients, which is manifested by longer PFS and OS (6, 7, 31, 32). Additionally, M1-type macrophages exert antitumor immunity, which is related to a better prognosis of immunotherapy (33). The expression of chemokines, such as CXCL9 and CXCL10, was significantly increased in the IL-1-MT group. These inflammatory mediators recruit CD8+ T cells, DCs and NK cells into tumor tissues to further exert an antitumor immune response (34). For example, CD8+ T cells can secrete cytotoxic mediators (such as perforin, granzyme and TNF) (34), CD4+ T cells secrete IL-6 and IFN-gamma further activates other immune cells (35). Moreover, we found that the IL-1-MT group had higher BCR Shannon index values, signature of HR defects, IFN-gamma responses, immune scores, leukocyte fractions, lymphocyte infiltration, signature scores, macrophage regulation, Th1 cells and Th2 cells. Studies have shown that IFN-gamma can further regulate the expression of MHC-I molecules on the surface of tumor cells by activating STAT1. The GSEA results also suggested that the chemokine signaling pathway and NK cells mediate cytotoxicity, and the activity of the MHC signaling pathway in the IL-1-MT group was significantly higher than that in the IL-WT group. Additionally, the expression levels of genes related to antigen processing and presentation and cytotoxicity were significantly higher in the IL-1-MT group than in the IL-1-WT group. The above results all suggest that an inflammatory immune environment forms in the tumors of IL-1-MT COAD patients, which may be a potential mechanism for these patients to have favorable clinical benefits after receiving ICIs.

The higher immunogenicity of the immune microenvironment in the IL-1-MT group may lead to a better prognosis after receiving ICIs. Studies have shown that higher immunogenicity can promote TIL levels in the TME (36–38). The TMB is a more reliable biomarker for predicting the efficacy of ICIs. A higher TMB is associated with better prognosis of immunotherapy (22, 39), and studies have shown that the NAL may be more accurate than the TMB in predicting the efficacy and prognosis of immunotherapy (11, 40). In addition to the TMB and NAL, the DDR pathway plays an important role in maintaining the stability of the body’s DNA (9, 10, 41). The increase in genomic instability is the result of mutations in the DDR pathway and further increases the TMB and NAL, ultimately leading to an increase in the infiltration of TILs in the TME (6). Studies have shown that the treatment of advanced metastatic bladder cancer patients with mutations in the DDR pathway with ICIs has significantly improved clinical benefits (10). Additionally, another pan-cancer study showed that patients with co-mutations in the DDR pathway have significantly longer survival times than those without co-mutations in the DDR pathway (9). Our results also suggest that the IL-1-MT group has a significantly increased number of mutations in the TMB, NAL, and DDR pathways. This increased immunogenicity may be the biological basis of why IL-1-MT COAD patients receiving ICIs have a better clinical prognosis. However, there are still some limitations. First, targeted sequencing (MSK-IMPACT) was used in the ICI-treated COAD cohort to detect somatic mutations, and targeted sequencing provides fewer gene mutations than WES; second, the ICI-treated cohort lacked transcriptomics, copy number variation (CNV), proteomics data and data related to the tumor evolution; therefore, the association between IL1-MT signaling and the prognosis of COAD patients treated with ICIs could not be further explored. Thus, we can only use the TCGA-COAD to explore the association between the IL1-MT and prognosis of COAD patients treated with ICIs based on multi-omics analysis; third, in future research, molecular and animal experiments are needed to further verify our results. Therefore, more studies involving larger samples and diverse ethnic groups are still needed for subsequent analysis and verification.

## Conclusions

In this study, IL-1-MT was found to be an independent predictor of good prognosis for COAD patients receiving ICIs. IL-MT COAD patients had a significantly prolonged OS. Additionally, IL-1-MT was associated with significantly increased immunogenicity, numbers of activated immune cells, inflammatory factors and immune response-related scores.

## Data Availability Statement

The original contributions presented in the study are included in the article/**Supplementary Material**. Further inquiries can be directed to the corresponding authors.

## Author Contributions

Conceptualization, QW, JLX, and YB. Formal analysis, XZ, YL, JX, CY, and YW. Visualization, XZ, YL, JX, and YW. Writing–original draft, XZ, YL, JX, and YW. Writing–review & editing, QW, JLX, YB, XZ, YL, JX, CY, and YW. All authors contributed to the article and approved the submitted version.

## Funding

This work was supported by grants from the Key research and development project of science and technology department of Sichuan province (2021YFS0128).

## Conflict of Interest

The authors declare that the research was conducted in the absence of any commercial or financial relationships that could be construed as a potential conflict of interest.

## Supplementary Material

Supplementary Figure 1 Lollipop plot shows the distribution of MYD88, IRAK4 and TRAF6 mutations in the TCGA-COAD cohort.

Supplementary Figure 2 The heatmap showed the difference of IL-1 signaling related genes between the IL-1-MT and IL-1-WT group.

Supplementary Figure 3 The comparison of the difference of the expression some genes with pro-inflammatory activity (such as IL1RN, IL1R2, IL1B, IL1R1, IL1RAP, IL1A) between the IL-1-MT and IL-1-WT group.
