# Supplementary figures and images for: Analysis of Interleukin-1 Signaling Alterations of Colon Adenocarcinoma Identified Implications for Immunotherapy
Source: Front Immunol. 2021 Jul 23;12:665002. doi: 10.3389/fimmu.2021.665002 (PMC8344046; doi:10.3389/fimmu.2021.665002)

**A**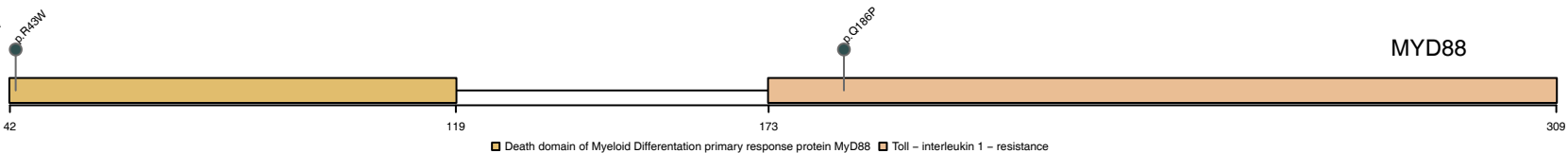**B**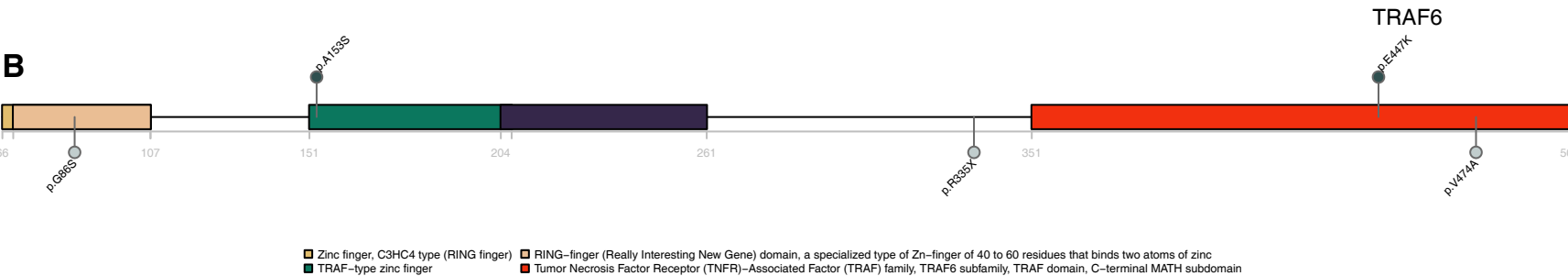**C**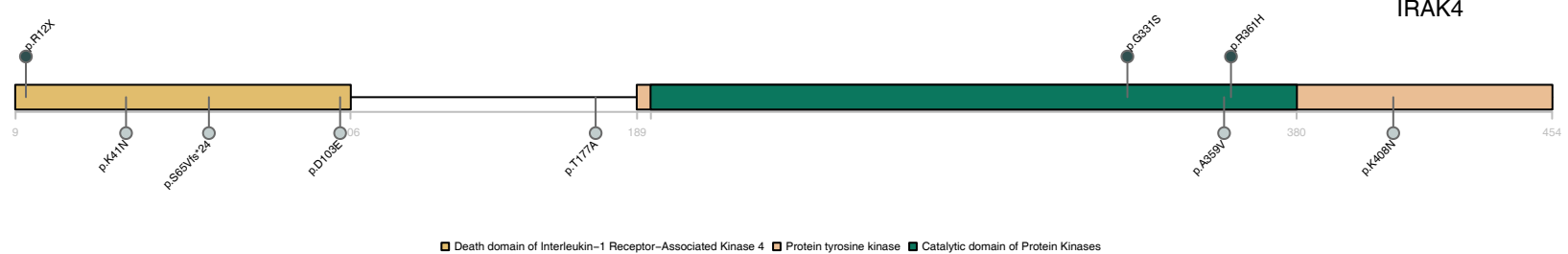

Supplement: Supplementary Figure 1 — Lollipop plot shows the distribution of MYD88, IRAK4 and TRAF6 mutations in the TCGA-COAD cohort. [file Image_1.pdf]

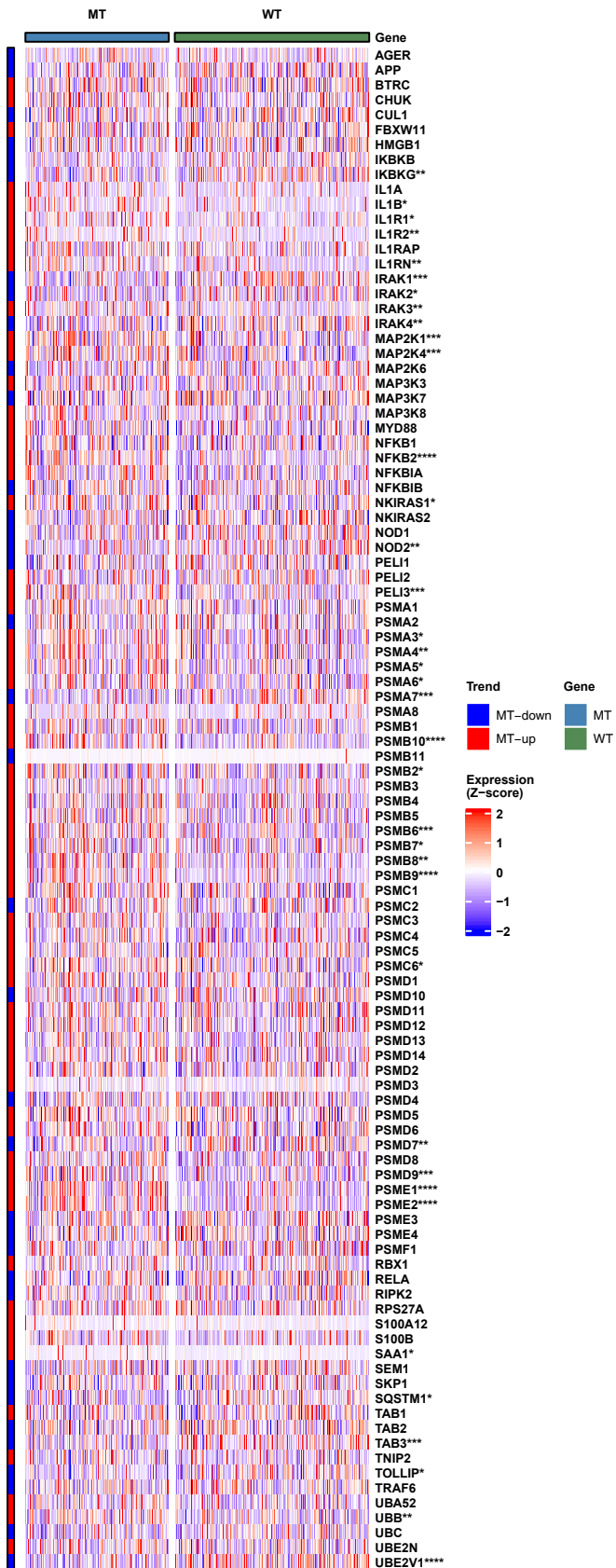

Supplement: Supplementary Figure 2 — The heatmap showed the difference of IL-1 signaling related genes between the IL-1-MT and IL-1-WT group. [file Image_2.pdf]

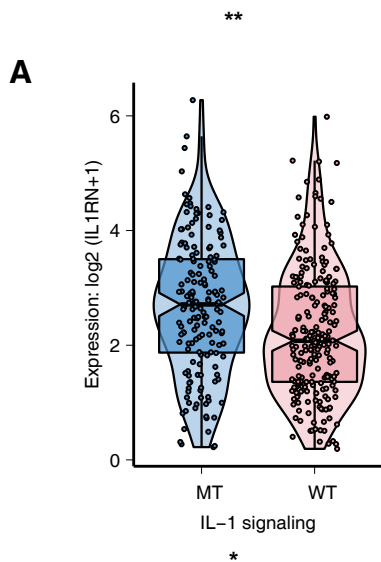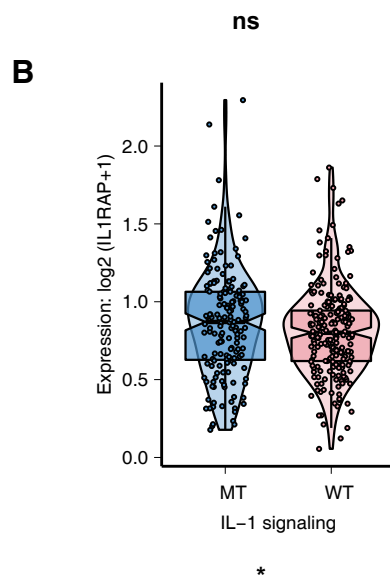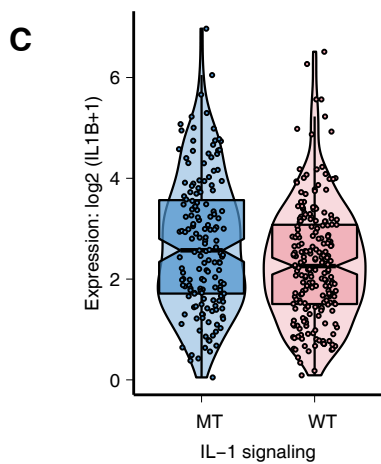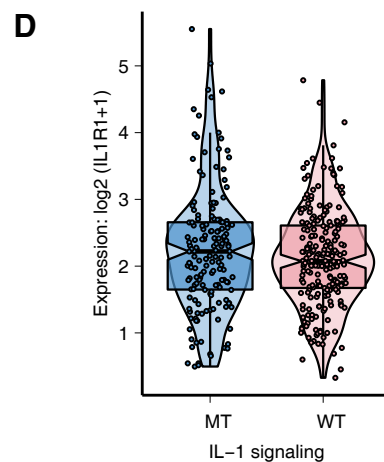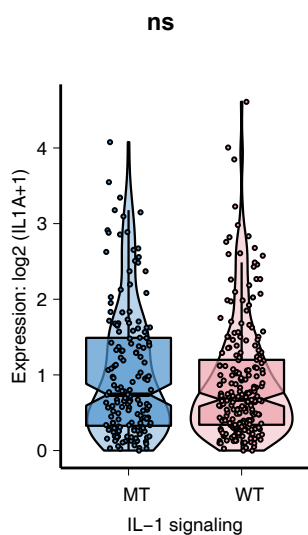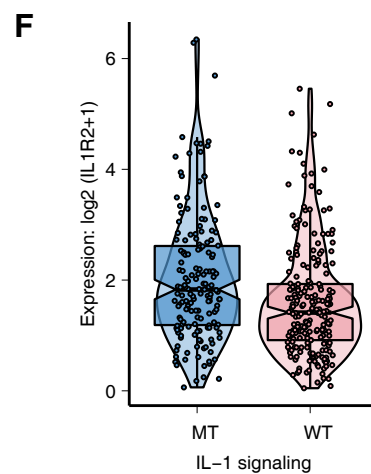

Supplement: Supplementary Figure 3 — The comparison of the difference of the expression some genes with pro-inflammatory activity (such as IL1RN, IL1R2, IL1B, IL1R1, IL1RAP, IL1A) between the IL-1-MT and IL-1-WT group. [file Image_3.pdf]
